# Beliefs about medications, health anxiety, and educational impact on treatment adherence in type 2 diabetes: A cross-sectional study

**DOI:** 10.1097/MD.0000000000043930

**Published:** 2025-08-15

**Authors:** Betülay Kiliç, Merve Nur Öz

**Affiliations:** a University of Health Sciences Turkey, Gulhane Faculty of Nursing, Ankara, Turkey.

**Keywords:** beliefs, diabetes mellitus, health anxiety, medication adherence

## Abstract

Low medication adherence is one of the leading causes that affect glycemic control and the achievement of target levels for type II diabetes mellitus (T2DM). It is hypothesized that negative beliefs about medications and high health anxiety may reduce adherence, while positive treatment beliefs could enhance it. This study aims to assess the impact of treatment beliefs and health anxiety on medication adherence among individuals with T2DM. This cross-sectional study was conducted with 356 T2DM patients. The study was conducted between July 2022 and January 2023 in a state hospital in Turkiye. Data were collected using the Morisky Green Levine Medication Adherence Scale (MGLS), the beliefs about medicines questionnaire [BMQ-Turkish translation (BMQ-T)] and the health anxiety inventory (HAI). According to the study’s findings, 42.7% of the participants adhered to the treatment. The mean age of the participants was 60.64 ± 12.13 years. It was found that with a low level of education, namely illiterate patients (β = 1.625, *P* = .003) and primary school graduates (β = 0.995, *P* = .008), the BMQ-T subscales of specific necessity (β = −0.340, *P* = .016) and general harm (β = 0.445, *P* = .001) had an independent predictor effect on medication adherence scores. Health anxiety was not found to affect treatment adherence (*P* = .947). Based on the findings of this study, low education levels, along with negative beliefs about the necessity of treatment and concerns about medication harm, are significant independent predictors of low medication adherence. To improve adherence, targeted educational interventions should be developed to address these misconceptions, focusing on patients with lower education levels. Additionally, policy changes should integrate structured educational programs and guidance on the benefits and safety of treatment, ensuring long-term adherence support for individuals with type 2 diabetes mellitus.

## 1. Introduction

Diabetes is a growing global health concern, with its incidence rising steadily worldwide.^[1]^ Chronic hyperglycemia in diabetic individuals can lead to serious complications, including diabetic retinopathy, nephropathy, and neuropathy.^[2]^ The burden of both acute and chronic complications imposes significant challenges not only on patients but also on healthcare systems and society as a whole.^[3]^ To mitigate this burden, individuals with diabetes must adopt necessary lifestyle changes and adhere strictly to their follow-up and treatment plans. However, poor medication adherence remains a critical issue, compromising treatment effectiveness and contributing to increased morbidity, mortality, and healthcare costs.^[1]^ In 2021, diabetes was responsible for 1.6 million deaths, with 47% occurring before the age of 70. Additionally, it contributed to 530,000 deaths due to kidney disease and accounted for 11% of cardiovascular-related deaths.^[4]^ Although diabetes is responsible for approximately 4% of global deaths, over 80% of these occur in low- and middle-income countries.^[5]^ Effectively managing this chronic disease remains a challenge, with non-adherence to antidiabetic medications being one of the most pressing concerns.^[6,7]^

Medication adherence refers to taking the correct dose at the right time as prescribed by a healthcare provider.^[8]^ Medication adherence can be affected by many interrelated factors, including patients’ beliefs about treatment, knowledge about the disease, access to health services, comorbidities, social support, and sociodemographic characteristics.^[9]^ Negative beliefs about medications are a substantial barrier to medication adherence. Conversely, patients with high beliefs about the necessity of drugs and low concerns about medications have been shown to have higher medication adherence.^[10,11]^

Adherence to treatment for diabetes helps maintain proper health and reduces diabetes-related complications. However, some psychological factors may compromise compliance and adherence in patients with diabetes mellitus.^[8,12]^ Health anxiety involves a person’s worry about their health or the fear that they may have a serious illness.^[13]^ In patients with diabetes, as in other chronic diseases, the rates of psychiatric symptoms and disorders are high. Studies have shown that the prevalence of anxiety in patients with type 2 diabetes is 30% to 46.5%.^[14,15]^ Diabetes distress leads to health anxiety because patients with chronic illnesses such as diabetes commonly experience fears of disease or symptoms recurring or worsening. It leads to lower adherence to treatment, fewer positive health behaviors, and increased medical costs.^[16]^ Health anxiety is higher among patients with diabetes.^[17]^

When patients are diagnosed with any disease, they often develop various beliefs about their condition. These beliefs constitute essential points of behavior for disease management and can affect compliance with treatment. To increase medication adherence in patients, it is first necessary to understand the problem’s size and scope and determine the factors that reduce treatment adherence.^[18]^ In the literature, the issue of compliance with diabetes treatment has been frequently discussed, and the effects of sociodemographic data and personal and disease-specific factors have been emphasized. However, studies examining patients’ beliefs about medication compliance with medication treatment are limited. Therefore, this study was planned to evaluate the adherence, belief level, and health anxiety of patients with T2DM by questioning the factors affecting their regular use of their medications

## 2. Materials and methods

### 2.1. Study design and setting

This study was conducted as a cross-sectional study. It was performed on participants with T2DM who applied to internal medicine outpatient clinics between July 2022 and January 2023. The study population consists of T2DM patients who visit a state hospital’s internal medicine outpatient clinic. According to the information obtained from the hospital, the number of patients who visited the hospital within a year is 4243. The sample size was calculated using the formula established by Salant and Dillman (1994):

n = N *t*2 *p q*/*d*2 (*N* − 1) + *t*2 *p q*

N: total population size; n: number of individuals to be sampled; p: frequency of occurrence of the studied event (probability of occurrence); q: frequency of nonoccurrence of the studied event (probability of nonoccurrence); *t*: theoretical value from the *t*-table corresponding to a given significance level; *d*: accepted margin of error for the occurrence frequency of the event.

The sample size was calculated using Salant and Dillman formula for this non-homogeneous population with a 95% confidence interval and ± 5% sampling error, and the required sample size was n = 4243 (1.96)2 (0.5) (0.5)/ (0.5)2 (149 − 1) + (1.96)2 (0.5) (0.5) = 352 participants.^[19]^

The inclusion criteria were: Having a diagnosis of Type 2 Diabetes Mellitus (T2DM) according to the diagnostic criteria of the American Diabetes Association^[20]^; using at least one antidiabetic medication (including oral antidiabetics such as biguanides, sulfonylureas, DPP-4 inhibitors, or insulin therapy) for at least 6 months before the study; being 18 years or older; having the ability to speak, read, and write in Turkish; providing informed consent to participate in the study. The exclusion criteria were: having a diagnosis of psychiatric disorders; having a cognitive impairment that prevents understanding the questionnaires and assessments; having a concurrent terminal illness or being clinically unstable; being unable to provide informed consent; being unable to communicate in Turkish. Since the study utilized questionnaires and assessment tools in Turkish, only individuals proficient in Turkish were included to ensure the reliability of the research. To ensure participants’ accurate understanding of the questionnaires and assessment tools, the researcher provided verbal explanations and support during data collection when needed. This approach was particularly adopted to facilitate the participation of individuals with low literacy levels. Ultimately, a total of 361 participants with T2DM were enrolled in the study. Five participants were excluded from the analysis due to incomplete questionnaires. Consequently, the analysis was limited to the data of the remaining 356 participants.

### 2.2. Data collection tools

Data were collected using the “Personal Information Form,” “Morisky-Green-Levine Medication Adherence Scale,” “health anxiety inventory,” and “Beliefs about Medicines Questionnaire-Turkish translation.

Participant assessment form: This form was used to collect data on sociodemographic (age, gender, education level, marital and working status, socioeconomic situation) and clinical characteristics (duration of disease, drug-related adverse events, comorbidities, using non-medication methods, status of receiving information/education about treatment) of the participants.

Morisky Green Levine Medication Adherence Scale (MGLS): This 4-item dichotomous scale was developed by Morisky et al (1986) to determine the adherence of patients to medication regimens. The reliability and validity in Turkish were realized by Bahar et al (2014).^[21,22]^ Each item asks patients whether they exhibit a specific type of non-adherent behavior. For each item, a yes or no response is assigned a score of 1 or 0, respectively. The score of the MGLS can range from 0 to 4, and higher scores indicate lower adherence to medication. In this study, participants are categorized into 2 groups according to their scores: 0 = adherent; 1, 2, 3, 4 = non-adherent.^[23]^ The Cronbach alpha value of the MGLS adapted to Turkish by Bahar et al (2014) was reported to be.62.^[22]^

Beliefs about medicines questionnaire (BMQ): This scale was validated for the Turkish population [BMQ Turkish Translation (BMQ-T)] by Cinar et al (2016) and is used to assess patients’ perceptions and expectations about medications. The original BMQ was developed by Horne et al (1999).^[24,25]^ It consists of general and specific sections, with 2 subscales for each. The subscales of the BMQ-general section are general harm and general overuse, both consisting of 4 items. The general harm subscale assesses patients’ beliefs about the degree to which drugs are perceived as essentially harmful, while the general overuse subscale addresses views on how drugs are used by physicians. The BMQ-specific consists of 2 subscales, specific necessity and specific concern, with 5 items each that assess patients’ beliefs about the necessity of prescribed medication for controlling their disease and their concerns about the potential adverse consequences of taking it. The higher scores of each section indicate stronger belief in the concept of that section. The Cronbach alpha value of the BMQ-T adapted to Turkish by Cinar et al (2016) was reported to be (specific necessity = 0.81; specific concerns = 0.67; general harm = 0.67; general overuse = 0.65).^[24]^ In the present study, the Cronbach alpha value of the scale was found to be (specific necessity = 0.79. specific concerns = 0.68; general harm = 0.72; general overuse = 0.54). The values obtained in the Specific Concern (α = 0.68) and especially the general overuse (α = 0.54) subdimensions indicate weak internal consistency as they fall below .70. Possible reasons for this include the low number of items in the subdimensions, the impact of cultural differences on perceptions regarding medications, and the heterogeneous views of individuals on these topics. Since Cronbach alpha coefficient is sensitive to the number of items, such low values can be expected, particularly in short subscales.^[26]^ The Cronbach alpha values for both subdimensions were found to be similar to those in previous studies in the literature.^[24]^ Nevertheless, these subdimensions were retained in the analyses in order to preserve the structural integrity of the scale and to ensure comparability with the literature.

Health anxiety inventory (HAI): The HAI is an 18-item, self-report scale developed by Salkovskis et al (2002). Each item is scored between 0 and 3, and the total score ranges from 0 to 54.^[27]^ Higher scores indicate higher health anxiety. The HAI has 2 subscales, named “Hypersensitivity about Structural and Physical Symptoms” and “Anxiety and Fear of Illness.” The validity and reliability of the HAI for use in Turkey were assessed by Aydemir et al (2013). The Cronbach alpha value of the HAI adapted to Turkish by Aydemir et al (2013) was reported to be 91.^[28]^ In the present study, the Cronbach alpha value of the scale was found to be .95.

### 2.3. Statistical analysis

Data were analyzed using SPSS 24.0 (IBM, Armonk). Continuous variables were expressed as mean ± standard deviation (if normally distributed) or median [Interquartile Range (IQR) (Q1-Q3)], (if non-normally distributed), and categorical variables were expressed as numbers and percentages. The compatibility of the continuous data with a normal distribution was examined using the Kolmogorov–Smirnov test. Pearson Chi-square test was used to compare categorical dependent and independent variables. While the data were not normally distributed, the Mann–Whitney *U* test was used to compare scores between 2 groups. For the data that were normally distributed, comparisons between the 2 groups were assessed using the Independent Samples *t*-test. Multivariate binary logistic regression analysis was performed to identify potential risk factors associated with medication non-adherence. Variables included in the multivariate model were selected based on their statistical significance (*P* < .05) in univariate analyses. Prior to inclusion in the multivariate model, multicollinearity among independent variables was assessed using the variance inflation factor. Variables with variance inflation factor values >10 were considered to have significant multicollinearity and were excluded or adjusted accordingly. Logistic regression was conducted using the “Enter” method, whereby all selected variables were entered into the model simultaneously. Medication adherence status, as defined by the Morisky Green Levine Scale, was used as the binary dependent variable. Missing data were examined before analysis. All missing data were excluded, and only complete cases were used in the analysis. Sensitivity analyses were also performed to assess the impact of missing data on the results. For all the analyses, a *P*-value of <.05 was accepted to be statistically significant.

### 2.4. Ethical approval

This study was approved by the Institutional Review Board of the university (Session No:2022/9). Participants were provided with information about the objective of the study, privacy, and confidentiality.

## 3. Results

### 3.1. Sociodemographic and clinical characteristics

According to the current study, 42.7% of the participants adhered to the treatment. The mean age of the participants (228 females, 128 male) was 60.64 ± 12.13 years. When the participants’ adherence to treatment and sociodemographic and clinical characteristics were compared, it was found that participants’ adherence to treatment significantly increased with education level (t = 17.75, p = <.01). The treatment adherence status was not changed with any other variables (Table 1).

**Table 1:** Sociodemographic and clinical characteristics of the participants (n = 356).

Characteristics	All participants 356 (100.0)	Adherent 152 (42.7)	Non-adherent 204 (57.3)	Statistics	*P*-value
Age (yr)*	60.64 ± 12.13	59.42 ± 12.89	61.54 ± 11.48	−1.641†	.102
Gender‡					
Female	228 (64)	93 (61.2)	135 (66.2)	943§	.332
Male	128 (36)	59 (38.8)	69 (33.8)		
Marital status‡					
Married	290 (81.5)	122 (80.3)	168 (82.4)	252§	.616
Single	66 (18.5)	30 (19.7)	36 (17.6)		
Educational status‡					
Illiterate^a^	32 (9.0)	8 (5.3)	24 (11.8)	17.75§	**.001** a < b, a < c, a < d b < c, b < d‖
Secondary^b^	239 (67.1)	92 (60.5)	147 (72.1)		
High school^c^	50 (14.0)	30 (19.7)	20 (9.8)		
University and over^d^	35 (9.8)	22 (14.5)	13 (6.4)		
Socioeconomic situation‡					
Less income than expenditures	84 (23.6)	32 (21.1)	52 (25.5)	3.221§	.200
Income equals expenditures	232 (65.2)	98 (64.5)	134 (65.7)		
More income than expenditures	40 (11.2)	22 (14.5)	18 (8.8)		
Duration of disease‡					
0–5 year	132 (37.1)	60 (39.5)	72 (35.3)	6.130§	.105
6–10 year	104 (29.2)	35 (23)	69 (33.8)		
11–15 year	48 (13.5)	20 (13.2)	28 (13.7)		
16 year and over	72 (20.2)	37 (24.3)	35 (17.2)		
Number of drugs used‡					
1–2	250 (70.2)	103 (67.8)	147 (72.1)	2.189§	.335
3–4	93 (26.1)	41 (27)	52 (25.5)		
5 and over	12 (3.7)	8 (5.3)	5 (2.5)		
Comorbidity‡					
Yes	276 (77.5)	114 (75)	162 (79.4)	0.973§	.324
No	80 (22.5)	38 (25)	42 (20.6)		
Getting information/education about treatment‡					
Yes	286 (80.3)	124 (81.6)	162 (79.4)	0.259§	.661
No	70 (19.7)	28 (18.4)	42 (20.6)		
Experiencing drug-related adverse events‡					
Yes	89 (25.0)	32 (21.1)	57 (27.9)	2.204§	.138
No	267 (75.0)	120 (78.9)	147 (72.1)		
Using non-drug methods‡					
Yes	128 (36.0)	58 (38.2)	70 (34.3)	559§	.445
No	228 (64.0)	94 (61.8)	134 (65.7)		

Bold values indicate statistically significant differences at *P* < .05.

* Mean ± standard deviation.

† Independent samples *t*-test.

‡ n (%).

§ Pearson chi-square test.

‖ Post hoc analysis.

### 3.2. Comparison of the medication adherence status of participants and the beliefs about medicines questionnaire scores and health anxiety scores

In terms of comparing BMQ-T scores treatment adherence, it was found that specific necessity scores were lower in non-adherent participants and general harm scores were higher (*P* = .009, *P* = .001) [Median (Q1–Q3): 3.9 (3.2–4.4) vs 2.87 (2.25–3.6) vs 4.2 (3.6–4.6), 2.5 (2.0–3.0) respectively]. Conversely, no significant relation existed between specific concerns scores and General Overuse with treatment adherence (*P* = .459, *P* = .291). Regarding comparing health anxiety scores and treatment adherence, there was no significant relation between health anxiety and treatment adherence (*P* = .947) (Table 2).

**Table 2:** Comparison of medication adherence, beliefs, and health anxiety (n = 356).

BMQ-T	95% CI	Overall (n = 356)	Adherence (n = 152)	Non-adherence (n = 204)	Z	*P*
		Median (Q1-Q3)	Median (Q1-Q3)	Median (Q1-Q3)		
BMQ-T-specific necessity	3.77–3.94	4.0 (3.4–4.5)	4.2 (3.6–4.6)	3.9 (3.2–4.4)	−2.596	**.009**
BMQ-T-specific concerns	2.91–3.09	3.0 (2.4–3.6)	3.0 (2.4–3.6)	3.1 (2.4–3.6)	0.741	.459
BMQ-T-general overuse	3.05–3.20	3.25 (2.5–3.6)	3.0 (2.5–3.5)	3.25 (2.75–3.75)	1.055	.291
BMQ-T-general harm	2.67–2.86	2.75 (2.25–3.5)	2.5 (2.0–3.0)	2.87 (2.25–3.6)	3.541	**.001**

HAI		Overall (n = 356)	Adherence (n = 152)	Non-adherence (n = 204)	*Z*	*P*
Total	37.35–39.37	37 (31–45)	37.5 (31–46)	37 (31–44.75)	15.56	.947
Hypersensitivity about structural and physical symptoms	29.18–30.86	28 (24–35)	28 (23–36)	28.5 (24–34)	15.38	.903
Anxiety and fear of illness	8.08–8.61	8 (6–10)	8 (6–10)	8 (6–10)	16.06	.588

Data represented either as median (Q1–Q3), or as the frequency. Bold values indicate statistically significant differences at *P* < .05.

BMQ-T = Beliefs about Medicines Questionnaire-Turkish translation, HAI = health anxiety inventory, *Z* = Mann–Whitney *U*.

### 3.3. Multivariate analysis of risk factors associated with medication non-adherence

The results of the multivariate analysis that were significantly associated with medication non-adherence of the participants are shown in Table 3. T2DM patients with lower necessity beliefs (BMQ-T-specific necessity) about the valuable consequences of antidiabetic medications and those with high beliefs that medicines are harmful (BMQ-T-general harm) were more likely to be non-adherent [(OR = 0.71; 95% CI = 0.54–0.83) and (OR = 1.54, 95% CI = 1.20–1.98), respectively].

**Table 3:** Multivariate analysis of risk factors associated with medication non-adherence.

Covariate	β	SE	Wald	Odds ratio*	*P*-value	95% CI
BMQ-T-specific necessity	−0.340	0.141	5.848	0.712	**.016**	0.540–0.838
BMQ-T-general harm	0.445	0.127	11.800	1.546	**.001**	1.206–1.981
Illiterate	1.625	0.538	9.132	5.070	**.003**	1.770–14.562
Secondary	0.995	0.374	7.065	2.204	**.008**	1.299–5.631

Bold values indicate statistically significant differences at *P* < .05.

β = coefficient of predictor variables, BMQ-T = Beliefs about Medicines Questionnaire-Turkish translation, CI = confidence interval, SE = standard error.

* Enter method was used.

## 4. Discussion

Persistent hyperglycemia is a hallmark of diabetes mellitus, a chronic metabolic disease caused by decreased insulin secretion, resistance to insulin’s effects, or both.^[29]^ Medication adherence plays a vital role in managing blood glucose levels and preventing complications in individuals with diabetes.^[30]^ Therefore, identifying factors related to treatment adherence is critical. The current study aims to assess medication adherence, health anxiety, and the role of beliefs about medicines on medication adherence in T2DM patients.

This study found that as educational status increased, patients’ adherence to treatment significantly increased. Health anxiety was not found to affect medication adherence. Regression analysis revealed that specific necessity and general harm scores of BMQ-T and educational status were the most highly related to medication adherence. Therefore, according to the current study, academic status, the specific necessity, and general harm of medication are significant predictors of medication non-adherence in T2DM patients.

This study found that patients with a high school education or higher had better adherence to treatment. In the literature, there are varying results regarding the relationship between the educational level of diabetic patients and treatment adherence.^[18]^ While some studies suggest that education level has no significant effect on medication adherence,^[29]^ others indicate that higher education levels are associated with improved treatment adherence.^[30–33]^ This may be due to individuals better understanding their disease, seeking ways to prevent complications, and developing self-management skills. In our study, being uneducated and a primary school graduate affects treatment adherence because most patients do not understand the treatment regimen. To improve adherence, educational materials with visual aids and simple language, along with structured diabetes education programs, should be implemented. Additionally, one-on-one counseling and community-based support programs should be expanded to enhance health literacy.

The literature highlights the complex and sometimes contradictory nature of patient-related factors influencing adherence, particularly age, ethnicity, education, and personality traits. Similarly, findings on socioeconomic factors such as gender and marital status remain inconsistent.^[18]^ This study found that, apart from education, sociodemographic variables did not affect adherence to diabetes treatment. A study conducted in Turkiye indicated that cultural norms, gender roles, and societal expectations could shape health behaviors, with women facing more significant barriers to adherence due to caregiving responsibilities. At the same time, men may not fully utilize healthcare services due to societal perceptions of masculinity.^[34]^ Future research should consider these socio-cultural dimensions to develop targeted interventions addressing adherence disparities.

Most studies on medication adherence have concluded that negative beliefs about medications are a substantial barrier to adherence.^[18,34–36]^ For instance, a cross-sectional study conducted in Greece involving 1988 patients with cardiovascular and cardiometabolic disorders found that individuals with negative beliefs about their medications were approximately 3 times more likely to exhibit poor adherence.^[11]^ In this study, it was found that the decrease in the belief that antidiabetic drugs are necessary and the belief that drugs are harmful affect treatment adherence in diabetic patients. Our findings regarding the relationship between medication adherence and belief in the study are consistent with the findings obtained by other researchers. Jiang et al. (2024) found the necessity of antidiabetic drugs and beliefs that drugs are essentially harmful as factors affecting non-adherence to treatment in diabetic patients. Another study reported that diabetic patients who have positive beliefs about medications and the importance of antidiabetic drugs are more likely to comply with treatment, while diabetic patients who believe that diabetic drugs have adverse effects and think that drugs are harmful are more likely to have low medication adherence.^[37,38]^ Aikens and Piette (2009) stated that the perceived necessity of medications is associated with medical factors such as the number of comorbidities, medications, and insulin use. It was noted that the perception that drugs are harmful is related to psychosocial factors such as health literacy and insufficient information about drugs.^[39]^ The present study suggests that the low education level of most patients may affect their beliefs about the harmfulness of drugs. It is considered that providing information to patients about the effects of medications, their side effects, and diabetes would be the most productive way. These findings underscore the importance of addressing patient beliefs to enhance medication adherence in chronic disease management.

Health anxiety remains a relatively underexplored topic among individuals with diabetes. Although the literature consistently highlights the influence of psychosocial factors on treatment adherence, this study did not find a significant association between health anxiety and adherence. In contrast, a systematic review examining various psychosocial factors affecting medication adherence in individuals with type 2 diabetes identified health anxiety along with diabetes-related distress, general stress, concerns about medication, and cognitive dysfunction as one of the primary factors negatively influencing adherence. Anxiety, in particular, has been reported as a significant psychological determinant adversely affecting both self-care behaviors and medication adherence. Similarly, concerns about medicines are also cited as major psychosocial variables that reduce adherence.^[40]^ In a study by Claude et al investigating health anxiety among individuals with diabetes, 24.1% of the sample reported high levels of health anxiety. This was especially pronounced among younger individuals, women, those who were single, and those recently diagnosed with diabetes. Health anxiety was further associated with certain personality traits, fear of diabetes complications, lower adherence to dietary and exercise self-care routines, and decreased physical quality of life.^[41]^ Additionally, a cross-sectional study conducted in Saudi Arabia involving 449 individuals with type 2 diabetes found a significant negative association between levels of anxiety and depression and medication adherence. Although the instrument used did not specifically assess health anxiety, the presence of anxiety and depressive symptoms alone was shown to negatively impact adherence.^[42]^ In another study by Altintaş et al, individuals with lower blood glucose levels were found to have higher levels of health anxiety.^[43]^ The association between health anxiety and poor adherence is a critical finding with implications for patient well-being and quality of life, and it should be considered in the development of health policies. However, the lack of a significant association in this study may be explained by several methodological and cultural factors. Response biases, such as social desirability, may have led participants to underreport their health anxiety, especially in cultural contexts where expressing anxiety is stigmatized. Moreover, the relative homogeneity of the sample and culturally influenced variations in the perception and expression of health anxiety may have limited the variability necessary to detect a significant relationship.

This study has several limitations. First, medication adherence was assessed using a self-report method, which may have led to over- or under-estimating adherence. Participants may have over-reported adherence due to social desirability bias or recall bias. While more accurate adherence data could be obtained through objective measures like pill counts or drug monitoring systems, self-reporting remains a practical and cost-effective approach, especially for large-scale studies. Another limitation is the study’s cross-sectional design, which restricts our ability to draw causal inferences. A longitudinal design would provide more robust evidence of causal relationships over time.

Additionally, the study was conducted at a single hospital, which may limit the generalizability of the findings. Replicating this study across diverse healthcare settings would enhance the external validity of the results. The use of translated instruments, such as the BMQ-T and HAI scales, may have introduced cultural or linguistic biases, potentially affecting participants’ responses. Future research should investigate whether these biases influence medication adherence measurements. Lastly, excluding patients with cognitive impairments may have resulted in the omission of a significant portion of the T2DM population. This limitation should be considered when interpreting the findings, as it may impact the broader applicability of the results.

## 5. Conclusion

Given these findings, tailored educational programs should be developed for patients with lower education levels, focusing on disease management and the importance of medication adherence. Additionally, belief-targeting strategies such as cognitive-behavioral interventions or motivational counseling should be integrated into diabetes care to address misconceptions about the necessity and safety of medications. The non-significance of health anxiety suggests that other psychological factors may play a more prominent role in adherence. However, further research must confirm this and rule out potential methodological limitations. Future studies should employ longitudinal designs to examine changes in medication adherence over time and explore additional psychosocial factors influencing adherence. Addressing patients’ medication beliefs is crucial for improving adherence and glycemic control, ultimately leading to better health outcomes. System-level changes, such as routine adherence assessments and integrating adherence-enhancing interventions into standard diabetes care, should be prioritized to support long-term treatment success.

## Acknowledgments

We thank the patients for taking the time to fill in the questionnaires and for contributing to the study.

## Author contributions

**Conceptualization:** Betülay Kiliç, Merve Nur Öz.

**Data curation:** Merve Nur Öz.

**Formal analysis:** Betülay Kiliç.

**Investigation:** Betülay Kiliç, Merve Nur Öz.

**Methodology:** Betülay Kiliç.

**Resources:** Betülay Kiliç, Merve Nur Öz.

**Supervision:** Betülay Kiliç.

**Writing – original draft:** Betülay Kiliç, Merve Nur Öz.

**Writing – review & editing:** Betülay Kiliç.
